# A Vision-Based Self-Calibration Method for Robotic Visual Inspection Systems

**DOI:** 10.3390/s131216565

**Published:** 2013-12-03

**Authors:** Shibin Yin, Yongjie Ren, Jigui Zhu, Shourui Yang, Shenghua Ye

**Affiliations:** State Key Laboratory of Precision Measuring Technology and Instruments, Tianjin University, Tianjin 300072, China; E-Mails: shibinyin_1987@163.com (S.Y.); jiguizhu@tju.edu.cn (J.Z.); shouruiyang@tju.edu.cn (S.Y.); shhuaye@tju.edu.cn (S.Y.)

**Keywords:** self-calibration, industrial robot, visual sensor, TCP

## Abstract

A vision-based robot self-calibration method is proposed in this paper to evaluate the kinematic parameter errors of a robot using a visual sensor mounted on its end-effector. This approach could be performed in the industrial field without external, expensive apparatus or an elaborate setup. A robot Tool Center Point (TCP) is defined in the structural model of a line-structured laser sensor, and aligned to a reference point fixed in the robot workspace. A mathematical model is established to formulate the misalignment errors with kinematic parameter errors and TCP position errors. Based on the fixed point constraints, the kinematic parameter errors and TCP position errors are identified with an iterative algorithm. Compared to the conventional methods, this proposed method eliminates the need for a robot-based-frame and hand-to-eye calibrations, shortens the error propagation chain, and makes the calibration process more accurate and convenient. A validation experiment is performed on an ABB IRB2400 robot. An optimal configuration on the number and distribution of fixed points in the robot workspace is obtained based on the experimental results. Comparative experiments reveal that there is a significant improvement of the measuring accuracy of the robotic visual inspection system.

## 1. Introduction

In the modern manufacturing industry product quality control is of great significance to improve product quality, decrease rejection rates and for cost savings. Vision-based 3D (three-dimensional) inspection technology is widely applied in quality supervision for its advantages of non-contact operation, fast acquisition speed, low cost and good stability [1–3]. In order to meet the needs of efficient and accurate inspection, 3D visual inspection systems usually consist of two parts, the visual sensor and the orienting device. The orienting device is used to place the visual sensor in the position of the featured object. For industrial applications, it is preferable that the orienting device be flexible and controllable, so that the product quality inspection can be highly efficient and human-free. This leads to the idea of adopting an industrial robot as the orienting device [4]. The robotic visual inspection system is promising because it combines the industrial robot's high flexibility and the visual sensor's high accuracy. It has been extensively applied in the automotive industry and aircraft manufacturing industry [5,6].

Although robotic visual inspection systems have successfully completed repetitive tasks in assembly lines, there are still some critical challenges to be overcome before their application in advanced tasks such as robot off-line programming, precise robotic measurement and so on. One of the challenges is associated with the system accuracy. Although industrial robots generally have high repeatability, their accuracy is much worse [7,8]. The challenge, therefore, is how to improve and maintain the system's accuracy in different manufacturing environments. According to [9], nearly 90% of robot errors are due to the mismatch between the nominal kinematic model and the accurate model. The mismatch is caused by the kinematic parameter variation in the robot manufacturing and assembly process. Robot calibration is a cost-effective way to improve robot position accuracy because it identifies a more accurate functional relationship between the joint transducer readings and the position of end-effectors. In general, robot calibration could be divided into four steps: Modeling, Measurement, Identification, and Verification and Correction [10]. Many researchers have devoted efforts to this field for more than two decades. Different kinematic models [11,12] and identification algorithms [13–15] have been developed. A variety of measurement techniques have been employed for calibration tasks ranging from coordinate measuring machines and laser tracking interferometer systems to economical ball-bar and customized fixtures [16–18]. Robot calibration is easier to implement with these convenient devices, however, the data acquisition process is relatively time consuming and the resolution of the identified parameters is limited by the external measuring devices. Moreover, for the robotic visual inspection system in the industrial field environment, it's desirable that the system be capable of performing calibration without any expensive external apparatus or elaborate setups, which is the so-called system self-calibration.

Robot self-calibration generally utilizes redundant sensors or imposes certain constraints, such as straight line paths or plane constraints on the end-effector. Based on the robot kinematic close-loop equations and the robot joint angle data, the kinematic parameters could be identified. Khalil and Besnard [19] installed two orthogonally allocated inclinometers on a robot end effector to calibrate the Stewart platform. However, some kinematic parameters were not observable and the transformation between the tool and the robot could not be determined. Bennett and Hollerbach [20] have successfully performed self-calibration with passive endpoint constraints, using only the inherent joint sensors in the manipulator. This constrains the mobility of the platform and the errors of the locked passive joints could not be calibrated. The self-calibration concept has also been extended to calibrate robotic visual systems with a hand-mounted visual sensor. Gong [21] calibrated a robotic measurement system using its internal laser sensor based on distance measurements. This method avoids calibration of the robot base coordinates, shortens the error propagation chain and improves the robot accuracy significantly over a typical robot workspace. However, this approach has the following drawbacks: firstly, the method is based on the distance error measured by the visual sensor and the resolution of the identified parameters is restricted by the sensor accuracy; secondly, hand-to-eye calibration is needed before robot calibration, which still utilizes the robot nominal kinematic model, and errors in hand-to-eye transformation will inevitably be transferred to the identified parameters.

In this paper, a novel robot self-calibration approach is proposed for a robotic visual inspection system. A visual sensor is mounted on the robot end-effector serving as a tool. Then a robot Tool Center Point (TCP) is defined and calibrated using the sensor model. In order to calibrate the system, the robot is controlled to align the TCP to a fixed reference point in the robot workspace at different robot poses. With fixed point constraints, a systematic technique is proposed to formulate the misalignment errors with robot parameter errors and TCP position errors. By changing the position of the fixed point, the robot will get adequate movements in its workspace and the self-calibration can be performed in the whole volume of the robot. Verification experiments show that the presented approach has improved the accuracy of the industrial robot significantly. The unique feature of this approach is that it eliminates the need of calibrating the transformation from the world coordinate system to the robot base frame as well as the hand-to-eye transformation, so this approach shortens the error propagation chain further and makes the calibration procedures more convenient to implement. Moreover, no external measuring device or elaborate setup is adopted in the self-calibration process. Therefore, it is well suited for the online inspection systems in the industrial field where autonomy is a major concern.

The remainder of the paper is organized into the following four sections: Section 2 presents first an introduction to the robotic visual inspection system. Then it introduces the robot kinematic model and error model. Section 3 details the robot self-calibration algorithms, including robot TCP calibration and self-calibration based on fixed point constraints. Section 4 provides experimental results to illustrate the effectiveness and advantages of the proposed self-calibration method. A comparison between the robotic system before and after robot calibration is also performed in Section 4. Conclusions are given in Section 5.

## 2. Mathematic Models of Robotic Visual Inspection System

### 2.1. Measurement Principle of Robotic Visual Inspection System

**Figure 1.**
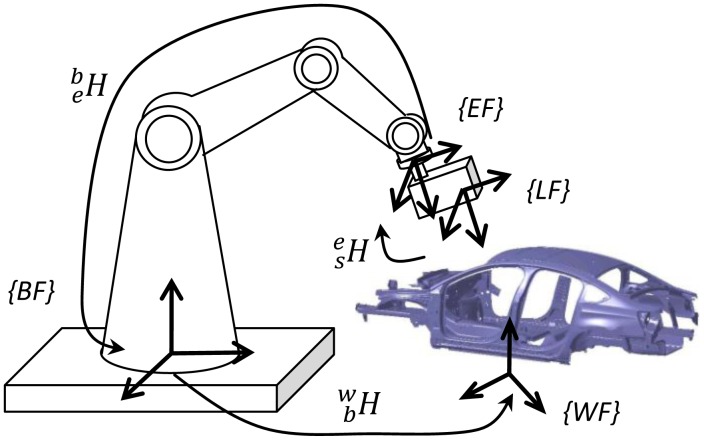
Schematic of robotic visual inspection system.

The robotic visual inspection system mainly consists of an industrial robot and a non-contact visual sensor. The industrial robot serves as the orienting device and locates the visual sensor to the designated featured points. As it has been extensively applied in the industrial field owing to its high-accuracy, high-speed and strong anti-interference, in the system of this work a line-structured laser sensor is adopted and mounted on the robot's end-effector via a metal rod. The coordinate systems of the robotic visual inspection system consist of robot base frame (BF), end-effector frame (EF), laser sensor frame (SF) and workpiece frame (WF), as shown in Figure 1.

The measured result of the visual sensor is usually transformed to the workpiece frame. For a visual point *P* on the work piece, the mapping relationship between the coordinate *P_w_* in the workpiece frame and *P_s_* in the sensor frame is expressed as follows:

(1) Pw=Tbw×Teb×Tse×Ps

where Tse denotes the transform matrix between the sensor frame and the robot end-effector frame, which is also called hand-to-eye relationship. Teb denotes the transform relationship between the robot end-effector frame and the robot base frame. It can be obtained from the robot forward kinematic model. Tbw is the transformation matrix between the robot base frame and the workpiece frame.

### 2.2. Robot Kinematic Model

As shown in Figure 1, the robot kinematic model relates the outputs of the robot joint sensors to the position and orientation of the robot end-effector. The serial link manipulator consists of a sequence of links. A mathematic representation based on the Denavit-Hatenberg (DH) convention is chosen to model the relationship between two consecutive joints. For two consecutive link frames *i*-1th and *i* th, four link parameters, namely joint angle *θ_i_*, link offset *d_i_*, link twist *α_i_* and link length *a_i_*, are used to represent the geometric relationship. The homogeneous transformation matrix is expressed as:

(2) Tii−1=[Cθi−SθiCαiSθiSαiaiCθiSθiCθiCαi−CθiSαiaiSθi0SαiCαidi0001]

where *Sθ_i_*, *Cθ_i_*, *Sα_i_* and *Cα_i_* represent sin *θ_i,_*, cos *θ_i,_*, sin *α_i_* and cos *α_i_* respectively. As the joints are rotational, only the *θ_i_* is the joint variable, *d_i_*, *α_i_* and *a_i_* are constants.

**Figure 2.**
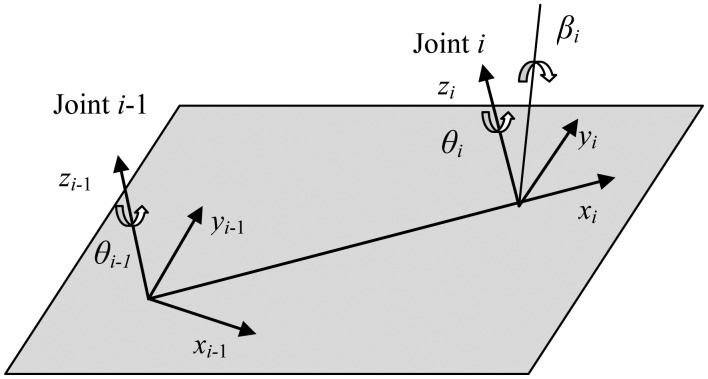
Model for two adjacent parallel joints.

As pointed out by Hayti [22], Equation (2) does not apply to those links with parallel or near parallel consecutive joint axes, because a small error in the alignment of the z*_i_* axis would cause a large error in parameters *θ_i_*, *d_i_* and *a_i_*. In order to overcome this problem, a small rotation of *β_i_* about *y_i_* axis is introduced while setting *d_i_* to zero, as shown in Figure 2. As the joints 2 and 3 of the researched robot in this work are parallel, the homogeneous transformation matrix has been improved as follows:

(3) Tii−1=[CθiCβi−SθiSαiSβi−SθiCαiCθiSβi+SθiSαiCβiaiCθiSθiCβi+CθiSαiSβCθiCαiSθiSβi−CθiSαiCβiaiSθi−CαiSβiSαiCαiCβidi0001]

where *Sθ_i_*, *Cθ_i_*, *Sα_i_*, *Cα_i_*, *Sβ_i_* and *Cβ_i_* represent sin *θ_i_*, cos *θ_i_*, sin *α_i_*, cos *α_i_*, sin *β_i_* and cos *β_i._*

For the serial robotic manipulator with *N* degrees of freedom, the transformation matrix from the robot end-effector frame to the robot base frame can be represented by:

(4) Teb=T1bT21T32⋯TNN−1=[RNTN01]

where *R_N_* and *T_N_* are the orientation and position of the robot end-effector in robot base frame described by the kinematic parameters. As reflected in Equation (3), errors in the kinematic parameters would result in deviation of the end-effector's pose (position and orientation) from the prediction. Furthermore, calibration of the hand-to-eye relationship and the transformation between the robot base frame and workpiece frame ( Tse and Tbw in Equation (1) respectively) generally involve robot movements. Without accurate kinematic parameters, these relationships cannot be determined in a high degree of precision. So there is a great demand for kinematic parameter identification and subsequent error compensation, especially in the high-precision robotic applications.

### 2.3. Error Model for Robot

The error model of the robot is used to investigate the relationship between positional, orientational error of robot end-effecor and the kinematic parameters error of robot joints [23,24]. Supposing that there are errors in the kinematic parameters of every joint, the actual transformation of the end-effector with respect to the robot base frame can be expressed as:

(5) Teb+ΔTeb=∏n=1N(Tnn−1+ΔTnn−1)

If the parameter deviations are small, the differential transformation can be represented by a linear function of the individual kinematic parameter deviation as follows:

(6) ΔTnn−1=∂Tnn−1∂θiΔθi+∂Tnn−1∂diΔdi+∂Tnn−1∂aiΔai+∂Tnn−1∂αiΔαi+∂Tnn−1∂βiΔβi

where *Δθ_i_*, *Δα_i_*, *Δa_i_*, *Δd_i_* and *Δβ_i_* are small variations of robot kinematic parameters.

Expanding Equation (5) and neglecting the high order terms, the differential transformation of the robot end-effector with respected to robot base frame can be approximated as in Equation (7):

(7) ΔTeb=∑i=1N(∂Teb∂θiΔθi+∂Teb∂diΔdi+∂Teb∂aiΔai+∂Teb∂αiΔαi+∂Teb∂βiΔβi)

where ∂Teb∂qi=T1b⋅T21⋅⋯⋅∂Tii−1∂qi⋅⋯⋅TNN−1 (*q_i_* denotes the kinematic parameters *θ_i_*,*d_i_*,*a_i_*,*α_i_*,*β_i_*).

Based on the robot differential kinematics equation ΔTeb=δT×Teb, we could rewrite the error model in matrix format as follows:

(8) $$\left(\begin{array}{c} \Delta D\\ \Delta \Theta \end{array}\right)=\left(\begin{array}{c} M_{\theta }\\ R_{\theta } \end{array}\right)\Delta \theta +\left(\begin{array}{c} M_{d}\\ 0 \end{array}\right)\Delta d+\left(\begin{array}{c} M_{a}\\ 0 \end{array}\right)\Delta a+\left(\begin{array}{c} M_{\alpha }\\ R_{\alpha } \end{array}\right)\Delta \alpha +\left(\begin{array}{c} M_{\beta }\\ R_{\beta } \end{array}\right)\Delta \beta$$

where Δ*θ* = [Δ*θ*_1_ Δ*θ*_2_ ⋯ Δ*θ_N_*]*^T^*, Δ*d* = [Δ*d*_1_ Δ*d*_2_ ⋯ Δ*d_N_*]*^T^*, Δ*a* = [Δ*a*_1_ Δ*a*_2_ ⋯ Δ*a_N_*]*^T^*, Δ*α* = [Δ*α*_1_ Δ*α*_2_ ⋯ Δ*α_N_*]*^T^*, and Δ*β* =[Δ*β*_1_ Δ*β*_2_ ⋯ Δ*β_N_*]*^T^*. Δ*D* and Δ*Θ* represent the positional and orientational errors. *M_θ_*,*M_d_*,*M_a_*,*M_α_*,*M_β_*,*R_θ_*,*R_α_* and *R_β_* are 3×*N* matrices of partial derivatives of the end-effector with respect to the kinematic errors.

In an alternative way, the relationship between partial positional and orientational deviation with respect to the kinematic parameter errors can be written in a compact form:

(9) ΔX=J×ΔP

where Δ*X* = [Δ*x* Δ*y* Δ*z* δ*x* δ*y* δ*z*]*^T^* represents robot end-effector's positional and orientational errors, Δ*P* = [Δ*θ* Δ*d* Δ*a* Δα Δ*β*]*^T^* depicts the robot kinematic parameter errors. *J* is the Jacobian matrix of identification defined in Equation (10). It outlines how each kinematic parameter error influences the robot positional and orientational accuracy.

(10) $$J=\left[\begin{array}{ccccc} M_{\theta } & M_{d} & M_{a} & M_{\alpha } & M_{\beta }\\ R_{\theta } & 0 & 0 & R_{\alpha } & R_{\beta } \end{array}\right]$$

## 3. Principle of the Vision-Based Self-Calibration Method

The robot self-calibration method presented in this paper is based on fixed-point constraints, in which the robot TCP is controlled to align to a reference point fixed in the robot workspace. The whole calibration process includes two procedures: robot TCP calibration and robot self-calibration.

### 3.1. TCP Calibration

Robot TCP is the center point of the tool ending defined in the robot program. It is a fixed point with respect to the robot end-effector and mainly used in the robot off-line programming. In the robotic visual inspection system, the line structured light sensor serves as a tool and robot TCP is defined in the sensor. Robot off-line programming is performed based on the defined TCP and the visual sensor is driven by the robot to inspect the featured points along the programmed trajectory.

The line-structured laser sensor works on the principle of triangulation and mainly consists of a laser stripe generator and a camera. When the laser stripe is projected on the surface of an object to be inspected, a contour line of laser is created and captured by the 2-D (2 Dimensional) Charge-coupled Device (CCD) camera. Then the shape is identified by extracting the laser stripe center and the point coordinate on the stripe can be calculated according to triangulation model.

**Figure 3.**
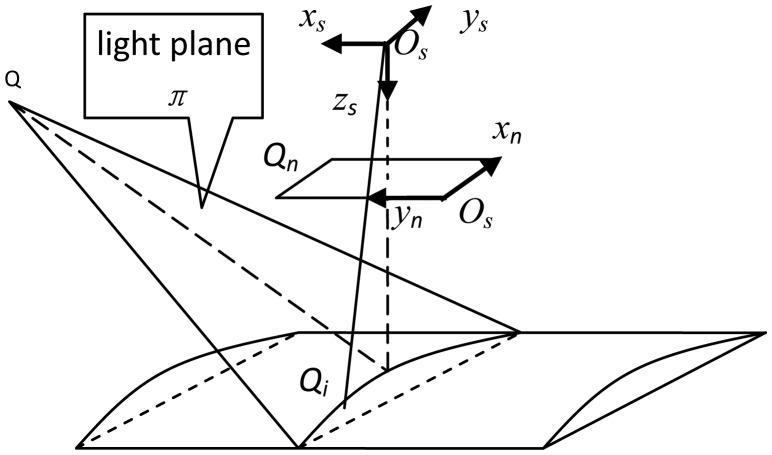
Mathematical model of line structure-line sensor.

As shown in Figure 3, *O_s_x_s_y_s_z_s_* is the laser sensor frame which is defined to be coincident with the camera coordinate and *x_n_O_n_y_n_* is image plane of the camera. In this paper, the TCP position is defined as the intersection point of the camera optical axis and the laser plane. When the laser sensor is mounted on the robot end-effector, the TCP is a fixed point with respect to the robot end-effector.

In this paper, a vision-based TCP calibration method is proposed. In this method, the robot is controlled to align the TCP to a fixed point at several different robot poses, that is to say, making the robot TCP position coincident with the fixed point. Assuming that *X_b_* is the coordinate of the fixed point *P* in the robot base frame, *R_i_* and *T_i_* are the orientation and position of robot end-effector. Line-structured laser sensor is fixed on the robot end-effector as a tool, assuming that *X_t_* is the TCP position relative to the robot end-effector. When the robot is controlled to align the TCP to the fixed point, the coordinates of the fixed point, referred to “measured position”, could be determined. If the alignment is performed in several different robot poses, the following equation can be obtained with the fact that position of the fixed reference point in the robot base frame is invariable:

(11) $$X_{b}=R_{1}\times X_{t}+T_{1}=R_{2}\times X_{t}+T_{2}=\cdots =R_{n}\times X_{t}+T_{n}$$

As TCP position is not determined, “measured position” cannot be obtained virtually. Subtracting each two adjacent equations in Equation (11), a matrix equation can be obtained in form of *AX* = *B*:

(12) $$\left[\begin{array}{c} R_{2}-R_{1}\\ \begin{array}{c} R_{3}-R_{2}\\ \vdots \end{array}\\ R_{n}-R_{n-1} \end{array}\right]X_{t}=\left[\begin{array}{c} T_{1}-T_{2}\\ \begin{array}{c} T_{2}-T_{3}\\ \vdots \end{array}\\ T_{n-1}-T_{n} \end{array}\right]\left(12\right)$$

As long as the coefficient matrix in Equation (12) is nonsingular, *X_t_* can be solved by means of least-squares method, that is:

(13) $$X_{t}={\left(A^{T}\times A\right)}^{-1}A^{T}\times B\left(13\right)$$

where: $A=\left[\begin{array}{c} R_{2}-R_{1}\\ \begin{array}{c} R_{3}-R_{2}\\ \vdots \end{array}\\ R_{n}-R_{n-1} \end{array}\right]$, $B=\left[\begin{array}{c} T_{1}-T_{2}\\ \begin{array}{c} T_{2}-T_{3}\\ \vdots \end{array}\\ T_{n-1}-T_{n} \end{array}\right]$.

The TCP calibration method presented here could also be formulated as minimizing the difference between any two of the “measured positions”. The calibration error of TCP position can be calculated as follows, which is also the least-squares fitting error:

(14) $$\delta =\left|\left[\begin{array}{c} R_{2}-R_{1}\\ \cdots \\ R_{n}-R_{n-1} \end{array}\right]X_{t}-\left[\begin{array}{c} T_{1}-T_{2}\\ \cdots \\ T_{n-1}-T_{n} \end{array}\right]\right|={\left(\sum_{i=1}^{n-1}{\left|(R_{i+1}-R_{i})X_{t}-T_{i}+T_{i+1}\right|}^{2}\right)}^{\frac{1}{2}}$$

### 3.2. Formulation of Self-Calibration Algorithm Based on Fixed Point Constraints

Based on the kinematic model in Equation (3) and the error model in Equation (9), a new robot self-calibration method can be formulated with the constraints of the fixed point. As the robot TCP is a fixed point with respect to the robot end-effector frame, if the robot is controlled to align the TCP to a fixed reference point in the robot volume from two different poses, the Cartesian positions of the fixed point in the robot base frame can be given as:

(15) Xi=Tebi×Xt,Xj=Tebj×Xt

where Tebi and Tebj are the transformations from robot end-effector to robot base frame which are calculated based on nominal kinematic model, *X_t_* is the TCP position calculated in Section 3.1. In this paper, *X_i_* and *X_j_* are referred to “nominal positions” and they should be equivalent nominally.

However, as the actual kinematic parameters of the robot may deviate from their nominal values, which are referred in Equation (15), there are errors between the “nominal positions” and the actual positions of the fixed point. Moreover, the TCP calibration method presented in Section 3.1 utilizes the robot forward kinematic model, so deviations would also exist in the TCP position *X_t_*. The actual position of the fixed point is given in Equation (16), and deviation between the actual position and the “nominal position” can be denoted as in Equation (17):

(16) XR=(Tebi+ΔTebi)(Xt+ΔXt),XR=(Tebj+ΔTebj)(Xt+ΔXt)

(17) XR−Xi=Tebi×ΔXt+ΔTebi×Xt+ΔTebi×ΔXt

Subtracting the deviations at two different robot poses and neglecting the second order terms, we have:

(18) (XR−Xj)−(XR−Xi)=Xi−Xj=(Tebj−Tebi)ΔXt+(ΔTebj−ΔTebi)Xt

In Equation (18), the deviations between the “nominal positions” of fixed point in two robot poses have been formulated with the TCP position errors and kinematic parameter errors. The deviation between the “nominal positions” is named “misaligned error” in this paper. According to the robot differential kinematics equation ΔTeb=δTTeb, we have:

(19) ΔTeb×Xt=δT×ebT×Xt=δT×Xb

Where: $\delta T=\left[\begin{array}{cccc} 0 & -\delta z & \delta y & \Delta x\\ \delta z & 0 & -\delta x & \Delta y\\ -\delta y & \delta x & 0 & \Delta z\\ 0 & 0 & 0 & 1 \end{array}\right]$ is the differential transformation, Xb=Teb×Xt=[b1b2b31].

In an alternative way, Equation (19) could be rearranged as follows:

(20) ΔTebXt=[0−δzδyΔxδz0−δxΔy−δyδx0Δz][b1b2b31]=[1000b3−b2010−b30b1001b2−b10][ΔxΔyΔzδxδyδz]=Q×ΔX

With the differential relationship given in Equation (9), Equation (20) could be written as:

(21) ΔTeb×Xt=Q×ΔX=Q×J×ΔP

Submitting Equation (21) into Equation (18) and rewriting Equation (18) in matrix form, we have:

(22) Xi−Xj=[Tebj−TebiQj×Jj−Qi×Ji][ΔXtΔP]

Let Tebj−TebiQj×Jj−Qi×Ji], *V_ij_* = *X_i_*−*X_j_*, $\Delta Y=\left[\begin{array}{c} \Delta X_{t}\\ \Delta P \end{array}\right]$ and suppose we have m (m > 4 × N, N:robot degrees of freedom) TCP alignment operations, then we obtain the following matrix equation:

(23) $$V=\left[\begin{array}{c} V_{ij,1}\\ V_{ij,2}\\ \vdots \\ V_{ij,m} \end{array}\right]=\left[\begin{array}{c} U_{ij,1}\\ U_{ij,2}\\ \vdots \\ U_{ij,m} \end{array}\right]\;\left[\begin{array}{c} \Delta X_{t}\\ \Delta P \end{array}\right]=U\times \Delta Y$$

In Equation (23), the kinematic parameter errors and TCP positional errors are the parameters to be identified. A Singular Value Decomposition (SVD) method, which is a rapid and computationally efficient algorithm, can be used to solve the matrix equation as:

(24) $$\Delta Y=(E\times S^{+}\times F^{T})V$$

where *E*, *F* are the right and left singular matrix of *U*, and *S*^+^ is the singular value matrix.

Updating the nominal kinematic parameters after the error identification, we can calculate the error for the robot self-calibration as follows:

(25) ε=(∑i=1n−1∑j=i+1n(Tebj×Xt−Tebi×Xt)T⋅(Tebj×Xt−Tebi×Xt))12

where Tebi and Tebj are the robot end-effector orientations and positions computed with the updated kinematic parameters.

**Figure 4.**
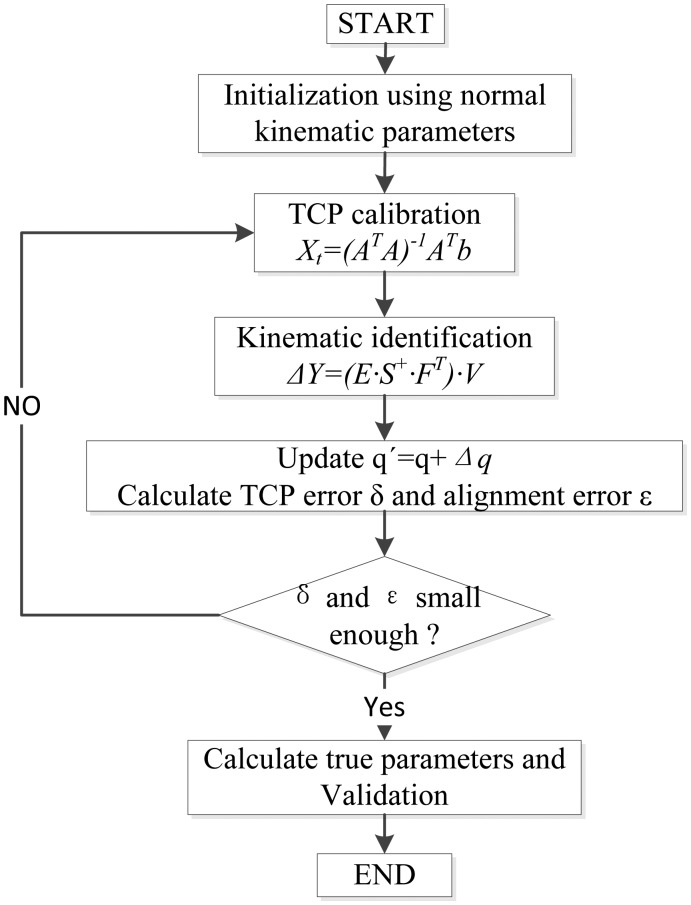
Flow chart of the robot self-calibration algorithm.

The purpose of robot self-calibration is to identify a more accurate kinematic model for the robot, with which the position and orientation of the end-effector can be predicted more accurately. Based on the kinematic model, the calibration model and the parameter identification techniques proposed in the preceding sections, the errors for kinematic parameters can be determined. The procedures for robot self-calibration are outlined by the flow chart shown in Figure 4. It can be seen from the chart that the self-calibration process is an iterative process. Errors for robot TCP position are also included in the system error model. Suitably small thresholds are selected for the TCP calibration error δ and kinematic parameter calibration error ε during the iterative computations. When the errors become less than the thresholds, the iteration will stop. As shown in the flow chart, the identified kinematic parameters are used to calibrate the TCP position again, so that the accuracy for the robot TCP position and kinematic parameters will improve step by step, which would be a virtuous promotion process.

Moreover, the fixed-point constraints-based calibration method identifies the robot kinematic parameters, and makes neither robot base calibration nor hand-to-eye calibration necessary. This blocks the estimated errors propagating from base-frame and hand-to-eye calibrations to the robot self-calibration procedure, and makes the parameter error identification more accurate. Note that the laser sensor is not used for measurement in the calibration process, so the accuracy of the sensor will have no impact on the error identification. In fact, the vision-based self-calibration method presented in this work could be implemented even with an un-calibrated line-structured laser sensor or just a camera and a laser stripe emitter fixed on the robot end-effector.

## 4. Experiments and Discussion

### 4.1. Experimental Setup

**Figure 5.**
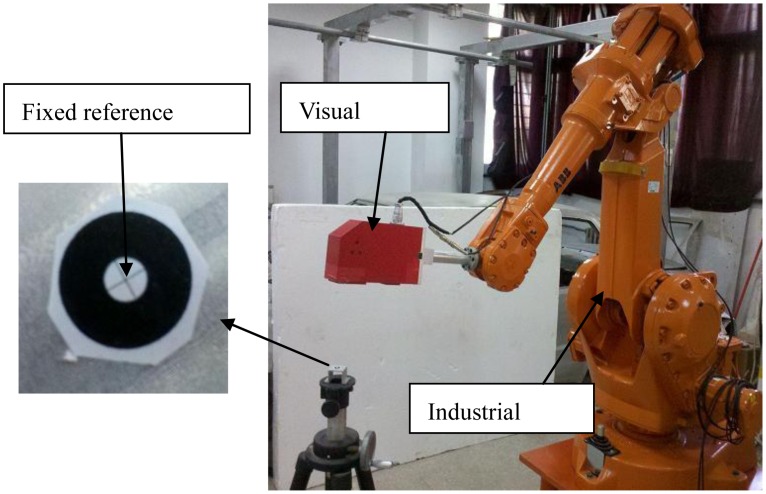
Experimental setup for robot self-calibration.

In order to verify the proposed self-calibration method, an experimental robotic visual inspection system is built. It consists of an ABB IRB2400 industrial robot, a line-structured laser sensor and a calibration target as shown in Figure 5. With high integration of state-of-art laser technology, electronic imaging and image processing technology, the laser sensor has an accuracy of 0.05 mm. The nominal value of the robot kinematic parameters is shown in Table 1. For the robot self-calibration setup, a sophisticated crosshair calibration target is positioned in the robot volume and its cross-center is referred to the fixed point constraint.

**Figure 6.**
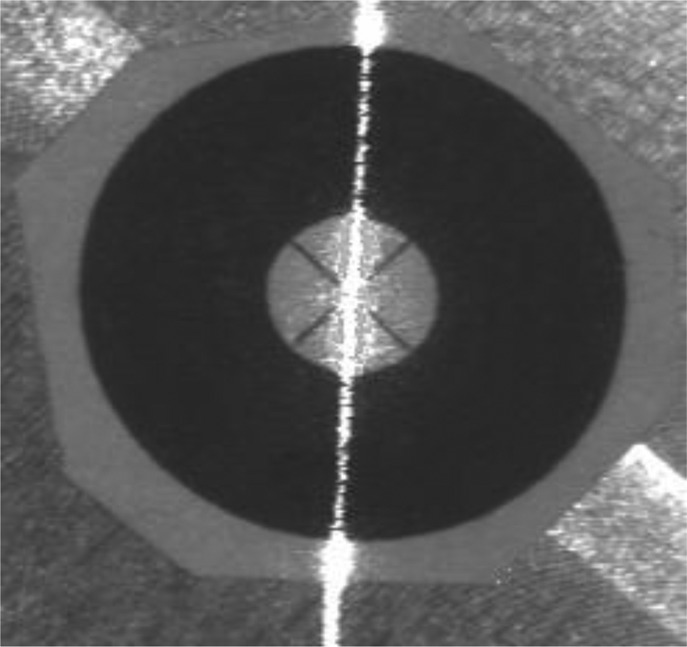
Alignment of the TCP with the fixed point.

During the calibration, the robot is controlled to align the robot TCP to the cross-center of the crosshair target at different robot poses. The purpose of the TCP alignment is to make the position of the robot TCP coincide with the fixed point. The TCP position in this paper is defined at the intersection point of camera optical axis and the laser plane. Based on the perspective projection principle of camera, all points on the optical axis will be imaged into the principle point on the image plane, which is determined in the camera intrinsic parameters calibration. When the image of the laser stripe also passes through the principle point, the principle point is the image position of TCP. If the fixed point is also projected at the principle point, TCP is aligned with the fixed point in the physical world. Hence the judging criteria of the alignment between the TCP and the fixed point are that the images of both the fixed-point and laser stripe will be coincident to the principle point on image plane. Picture captured by the camera of the laser sensor is shown in Figure 6. In practical operation, we write an interface program in Visual C++ (VC) to extract the centerline of the laser stripe in real-time and to align the laser stripe to the principle point on the image plane efficiently and instantly. The center of the laser stripe is extracted based on the Gaussian curve approximation method [25]. The joint angles are saved to identify the kinematic parameter errors via the identification method presented in Section 3.2.

**Figure 7.**
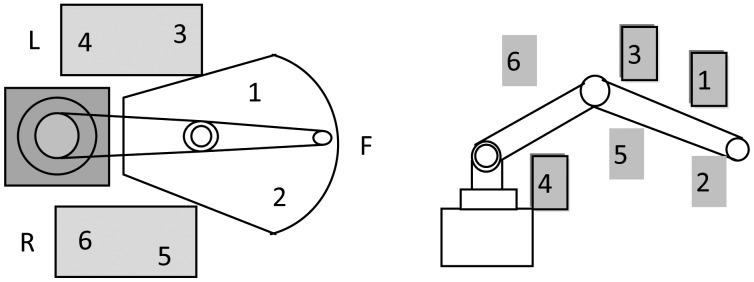
Locations of the fixed point in robot workspace.

Due to limited field of view of the camera, only a small part of the robot volume can be tested at any given position of the calibration target. To cover more robot volume, the calibration target must be placed at different heights and the robot workspace is divided into three local regions, namely right, front and left regions. In our experiment, the calibration target is positioned at high and low positions in each region, which are numbered and shown in Figure 7. The robot TCP is controlled to align to the cross-center at 10 robot poses at each location.

Meanwhile, there are many restrictions for the installation of the robotic visual inspection station on the manufacturing floor. It is noted that the distribution of the fixed point is not arbitrary and a minimal number of fixed points is expected. However, the number of fixed point as well as their distribution in the robot workspace would have great impact on the efficiency of the self-calibration procedure and effectiveness of error compensation. Hence, it's of great significance to learn the influence of the number and distribution of the fixed points prior to obtain an optimal configuration of the fixed points.

### 4.2. Result and Discussion

As shown in Figure 4, robot self-calibration method presented in this paper is an iterative process. Before the robot calibrating itself, initial values of the TCP position must be pre-determined. We align the robot TCP to the fixed point at NO.1 location at 4 different poses with the method presented in Section 3.1, and identify the TCP position relative to the robot end-effector: $X_{t\_\text{initial}}={\left[\begin{array}{ccc} 279.667 & -2.015 & 362.650 \end{array}\right]}^{T}$

**Figure 8.**
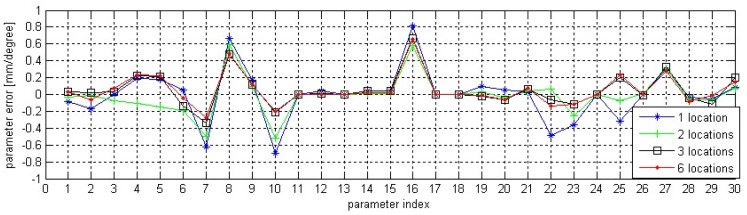
Comparison among the parameter sets from four different data sources.

After initial TCP calibration, the robot self-calibration is performed based on the calibration method in Section 3.2 and the data acquisition strategy in Section 4.1. In order to demonstrate how the number and distribution of the fixed points in the robot volume will influence the self-calibration, we identify the errors of kinematic parameters based on data from different locations (regions) and then compare the error compensation results. Since many of the experiments have similar results, only four sets of experimental data, with calibration and testing, are presented here for brevity, which are data from NO.1 location, data from NO.1 and NO.2 locations, data from NO.2, NO.3 and NO.6 locations and data from all the six locations. Note that there are total 33 parameter errors (30 kinematic parameters plus 3 TCP position parameters) in Equation (23) to be identified. According to Equation (18), TCP alignments at each two robot postures will provide one misalignment error, and each misalignment error has three components. Therefore, at least 11 misaligned errors, from at least six TCP alignments, are required for a unique solution to the 33 parameter errors. In this paper, the robot has been moved in 10 different poses at each location, so there are 45 misaligned errors at each location. Based on the misaligned errors from the four data sources, we could identify four sets of kinematic parameter errors. Comparison among them is shown in Figure 8.

In Figure 8, the 30 parameters are *α_1_* to *α_6_*, *a_1_* to *a_6_*, *d_1_* to *d_6_*, *θ_1_* to *θ_6_*, *β_1_* to *β_6_*. From Figure 8, we could find that there is divergence among the four parameter sets, especially for the parameter sets identified with data from one location and two locations. This is because the robot moves in a relatively limited workspace and the joint data is acquired in a concentrated area. With more TCP alignments in more locations, the estimation error gradually decreases. This could be seen from the parameter set identified based on the joint data from three locations, one fixed point distributed in each region. The minor divergence between the parameter sets identified with data from three locations and six locations also hints that the parameter identification algorithm can be further convergent and effective with more appropriate data samples. The identified kinematic parameter errors based on the data from three locations are given in Table 2 and the updated TCP position after one iteration is: $X_{t\_\text{updated}}={\left[\begin{array}{ccc} 280.215 & -2.377 & 361.319 \end{array}\right]}^{T}$.

**Figure 9.**
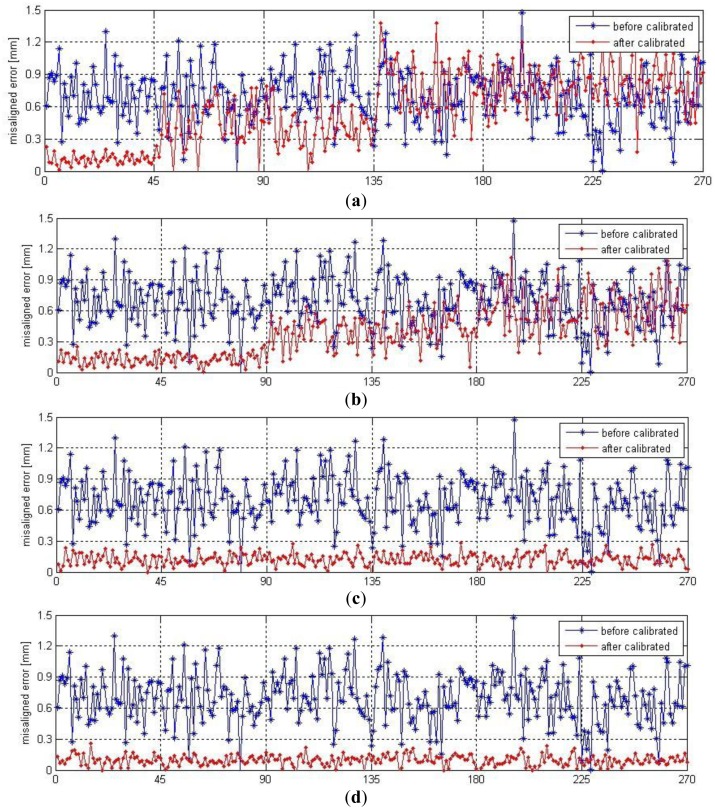
Misaligned errors compensation with four identified parameter sets. Error compensation with parameter identified based on data from: (**a**) location NO. 1, (**b**) locations NO. 1,2, (**c**) locations NO. 2,3,6, (**d**) all locations.

In order to verify the self-calibration approach and compare the effectiveness of the identified parameter sets, we use four sets of identified parameters to compensate for the misaligned errors from all the six locations. From the comparative result in Figure 9, we can see that with all the four parameter sets, the misaligned errors at the locations, whose data has been used to identify that parameter set, have all been compensated significantly. But the parameter sets estimated by one and two location are not so effective when they are used to compensate for the misaligned errors in the unoccupied regions. We note that the third parameter set estimated by data from Locations 2,3,6 has compensated for the misaligned errors at all the locations effectively. This is because all the robot joints have had adequate movements. The Figures 9c,d show that the third parameter set has similar effect with the parameter set estimated by data from all the locations. The mean values of the misaligned errors are 0.168 mm and 0.105 mm respectively. When applied on the manufacturing floor, three fixed points with a middle-low-high configuration distributed in the robot right, front and left regions would be effective and rational.

### 4.3. Verification

In order to verify the effectiveness of the calibrated kinematic parameters, an experimental system is setup to compare the measurement accuracy of the robotic inspection system with a laser tracker (Leica AT901 Laser Tracker with accuracy of ±15 μm + 6 μm/m). A sophisticated magnetic nest for the 38.1 mm spherically-mounted reflector (SMR) of the laser tracker is mounted on a scaffold which can move up and down freely. The scaffold has been placed at five different positions with different heights in each of the three regions of the robot workspace depicted in Section 4.1. At each scaffold position, the 38.1 mm SMR is mounted on the magnetic nest first and the center of the SMR is measured by the laser tracker. Then the SMR is replaced by a standard sphere with a diameter of 38.1 mm. Controlling the robotic system to scan the standard sphere and the sphere center could be found from the scan data. For further accuracy, bearing steel ball (G20 according to ISO 3290-2001) painted with white color is used as the standard sphere. The standard sphere was been first inspected on a CMM by sampling 20 points on the sphere surface and the sphericity (the maximum deviation of the points from the least-squares fitted sphere) of the spheres is found to be about 15 μm.

**Figure 10.**
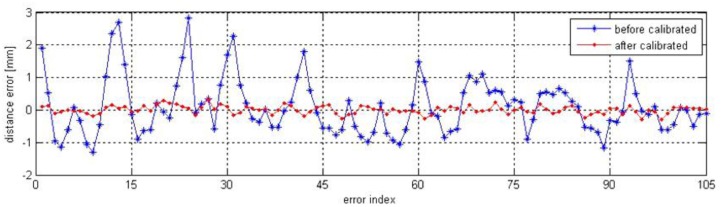
Distance error before calibration and after calibration.

The robotic visual inspection system scans the standard sphere according to the measuring model described in Section 2.1. According to Equation (1), hand-to-eye transformation Tse must be initially determined before the robotic system is applied for measurement. In this paper, we perform the hand-to-eye calibration based on the approach presented in [26]. With the nominal and the calibrated kinematic parameters in Table 2, two hand-to-eye transformations can be estimated. Likewise, the point cloud of a standard sphere can be transformed to the robot base frame based on the nominal and calibrated kinematic models. Then two groups of errors for sphere center distance are obtained from these two point clouds, which are distance errors before and after robot calibration. The comparison of the distance errors is shown in Figure 10. The maximum error has been reduced from 2.839 mm to 0.387 mm and the mean error has been reduced from 0.721 mm to 0.069 mm. Note that the mean value for the distance error is almost the same as the measurement accuracy of the laser sensor, which is 0.05 mm. The result of the verification confirms that the vision-based self-calibration method has significantly enhanced the overall performance of the robotic visual inspection system.

## 5. Conclusions

In this paper, a novel robot self-calibration approach is proposed to calibrate the kinematic parameter errors of a robotic visual inspection system based on fixed point constraints. Robot TCP is defined and calibrated based on the model of line-structured laser sensor, and then the robot TCP is controlled to align to points fixed in the robot workspace. There is no need to calibrate the transformation from the world coordinate system to the robot base frame as well as hand-to-eye transformation, which shortens the error propagation chain and increases the accuracy of the identified parameters. The effectiveness, correctness and reliability of the proposed method are proved via the experimental calibration and validation results with an ABB IRB2400 robot. The maximum value of the distance measuring error has been reduced from 2.839 mm to 0.387 mm. The comparison of compensation effectiveness of calibrated kinematic parameters from different number and distribution of the fixed points has indicated that the self-calibration approach is very effective. Moreover, the proposed method is well suited for robotic online inspection systems in the industrial field because no external measuring device or elaborate setup is needed in the whole self-calibration process. In the future, we will focus on improving the efficiency of the calibration procedures and promoting this robot self-calibration method in the industrial field.

## Figures and Tables

**Table 1.** Nominal value of kinematic parameters for IRB2400.

**Link No.**	***α*_i_/deg**	***a*_i_/mm**	***d*_i_/mm**	***θ*_i_/deg**	***β*_i_/deg**
1	−90	100	615	θ_1_	0
2	0	705	0	θ_2_–90	0
3	−90	135	0	θ_3_–θ_2_	0
4	90	0	755	θ_4_	0
5	90	0	0	θ_5_–180	0
6	0	0	85	θ_6_	0

**Table 2.** Identified values for kinematic parameters errors.

**Link No.**	**Δα_i_/deg**	**Δa_i_/mm**	**Δd_i_/mm**	**Δθ_i_/deg**	**Δβ_i_/deg**
1	0.035	−0.279	−0.017	−0.067	0.228
2	−0.060	0.482	0.026	0.071	0.024
3	0.075	0.114	0.654	−0.136	0.278
4	0.229	−0.202	0.032	−0.111	−0.082
5	0.237	−0.016	−0.025	0.045	−0.094
6	−0.047	0.027	0.091	−0.028	0.146
